# Mammographic Breast Density and Urbanization: Interactions with BMI, Environmental, Lifestyle, and Other Patient Factors

**DOI:** 10.3390/diagnostics10060418

**Published:** 2020-06-20

**Authors:** Nick Perry, Sue Moss, Steve Dixon, Sue Milner, Kefah Mokbel, Charlotte Lemech, Hendrik-Tobias Arkenau, Stephen Duffy, Katja Pinker

**Affiliations:** 1 London Breast Institute, Princess Grace Hospital, London W1U 5NY, UK; susanemilner@hotmail.co.uk (S.M.); Kefah.Mokbel@hcahealthcare.co.uk (K.M.); 2 Wolfson Institute, Queen Mary University of London, London EC1M 6BQ, UK; suemoss2@virginmedia.com (S.M.); s.w.duffy@qmul.ac.uk (S.D.); 3 HCA Healthcare UK, London NW1 6JQ, UK; mrstevecdixon@outlook.com; 4 Scientia Clinical Research, Sydney, Australia and Prince of Wales Hospital Clinical School, UNSW, Sydney NSW 2031, Australia; charlotte.lemech@scientiaclinicalresearch.com.au; 5 Sarah Cannon Research Institute UK and University College London, London W1G 6AD, UK; Tobias.arkenau@sarahcannonresearch.co.uk; 6 Department of Radiology, Breast Imaging Service, Memorial Sloan Kettering Cancer Center, New York, NY 10065, USA; pinkerdk@mskcc.org

**Keywords:** breast cancer risk, body mass index, breast density, mammography, urban

## Abstract

Mammographic breast density (MBD) is an important imaging biomarker of breast cancer risk, but it has been suggested that increased MBD is not a genuine finding once corrected for age and body mass index (BMI). This study examined the association of various factors, including both residing in and working in the urban setting, with MBD. Questionnaires were completed by 1144 women attending for mammography at the London Breast Institute in 2012–2013. Breast density was assessed with an automated volumetric breast density measurement system (Volpara) and compared with subjective radiologist assessment. Multivariable linear regression was used to model the relationship between MBD and residence in the urban setting as well as working in the urban setting, adjusting for both age and BMI and other menstrual, reproductive, and lifestyle factors. Urban residence was significantly associated with an increasing percent of MBD, but this association became non-significant when adjusted for age and BMI. This was not the case for women who were both residents in the urban setting and still working. Our results suggest that the association between urban women and increased MBD can be partially explained by their lower BMI, but for women still working, there appear to be other contributing factors.

## 1. Introduction

Mammographic breast density (MBD) has been recognized as an independent biomarker of breast cancer risk, with risk prediction of relevance to both primary and secondary prevention [1,2]. In the clinical setting, MBD is routinely assessed qualitatively by subjective radiologist review, which is prone to great intra- and inter-observer variability [3,4], whereas for study purposes, MBD is usually quantified in absolute or percentage terms. To overcome the limitations of qualitative assessments, computer-aided semi-automated and fully automated measurement approaches are now available and can be either 2-D area-based or 3-D volumetric-based methods for assessing MBD.

MBD is influenced significantly by age and body mass index (BMI) [5,6] and therefore, adjusting for these factors is important in studies of risk factors for breast cancer [7]. In addition, it has been shown that MBD is associated with lifestyle factors [8], socioeconomic status [6], and menstrual and reproductive factors [9]. Some studies have also shown an association of urban residence with increased breast density [10,11,12]. It has been hypothesized that this is due to lifestyle and environmental factors [10,12], and is associated with an increased risk of breast cancer with exposure to fossil fuel emissions and traffic fumes, which are said to have a weak estrogenic effect but may be inhaled in large volumes [13,14,15,16].

The aim of this study was to investigate the associations of various factors, including BMI, age, lifestyle, menstrual, and reproductive factors, as well as place of work and residence (urban vs. rural) with MBD and to compare the association of these factors with percent breast density and absolute volume of dense tissue. An additional aim was to compare MBD measurements obtained with an automated observer-independent quantitative breast density measurement system with qualitative MBD assessment by subjective radiologist review.

## 2. Materials and Methods

This prospective study was carried out at The London Breast Institute at the Princess Grace Hospital, an independent hospital providing specialist breast care services, including screening and diagnostic mammography. The study was reviewed by the Hospital Corporation of America (HCA) Healthcare UK Research Review Committee on behalf of the Princess Grace Hospital and was considered to be audit, not research, and therefore did not require specific ethics committee approval.

### 2.1. Study Sample

The study population consisted of 1144 women attending for full-field digital mammography (GE 2000D) at the London Breast Institute between late October 2012 and January 2013 and consenting to participate in this study. These represented approximately 85% of the total number of women attending for mammography during this period. Women were given a questionnaire at the time of attendance, together with a letter explaining the purpose of the study, and if willing to participate, they could complete the questionnaire at that time or return it later by post. It was made clear in the letter that women were welcome to discuss any further questions at the time with the technologist performing the mammogram. Completion of the questionnaire was therefore taken as consent to participate. Approximately half of the women were asymptomatic and attending for routine screening mammography, while the rest were symptomatic, with various complaints ranging from pain to the presence of a breast lump.

### 2.2. Questionnaire

The questionnaire included items on demographic information, lifestyle factors, menstrual and reproductive variables, and use of hormone replacement therapy (HRT) or oral contraceptives (OCs). Questions on past history of breast biopsy, cancer diagnosis, and family history of breast cancer were also included, together with information on the postcode of residence at the time of the most recent mammogram, previous postcode if the woman had moved within the past 5 years, postcode of place of work, and postcode of the previous place of work if this had changed within 5 years.

These postcodes were used to determine whether or not the woman was a resident and/or worked in the urban setting, i.e., London. Where the postcode of the place of residence was missing from the questionnaire (*n* = 56), information was taken from the patient records. Working in the urban setting was defined on the basis of the current place of work, excluding those with work type recorded as “homemaker” or “retired”, who would not be travelling to work.

Data on height and weight were used to calculate BMI and were available for 1098 women.

Data on smoking were used to calculate ‘pack-years’, using the formula, number of years smoked × number of cigarettes per day/20.

Data on menstrual periods were combined to determine menopausal status. Women reporting still having regular periods were classified as pre-menopausal, and those aged under 60 years reporting not having regular periods but having time since last period as less than 12 months were classified as peri-menopausal. Women reporting not having regular periods, and where either time since last period was more than 12 months or the woman was over the age of 60 years, together with those women over age 50 years where the current age was more than 1 year older than the age at last period, were classified as post-menopausal.

### 2.3. Breast Density Assessment

Where there was more than one mammography attendance recorded for a woman, the mammogram closest to the date of the questionnaire, based on patient age, was used to assess breast density.

**Figure 1:**
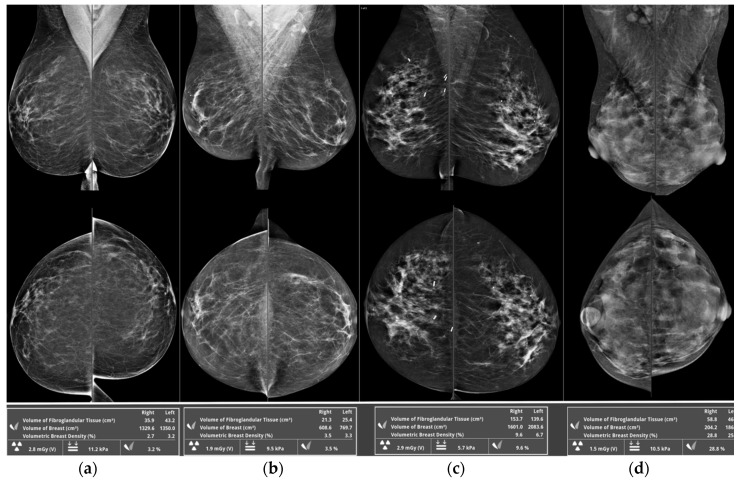
Qualitative mammographic breast density categories according to the fourth edition BI-RADS atlas (A = almost entirely fatty (**a**); B = scattered fibroglandular densities (**b**); C = heterogeneously dense (**c**); D = extremely dense (**d**)) with corresponding automated percent density measurements allocated by Volpara software into Volpara Density Grades (VDGs) (VDG 1 = 0.0–4.4% density, VDG 2 = 4.5–7.4%, VDG 3 = 7.5–15.4%, VDG 4 ≥ 15.4%).

Volumetric density for each mammogram was measured for each side and for each mammographic view using an automated observer-independent quantitative breast density measurement system (VolparaTM, Matakina). Percent breast density, absolute density (the absolute amount of fibroglandular volume), and total breast volume for each woman were calculated as averages for all views for the given attendance/study date. Automated percent density measurements were allocated by Volpara software into Volpara Density Grades (VDGs): VDG 1 = 0.0–4.4% density, VDG 2 = 4.5–7.4%, VDG 3 = 7.5–15.4%, VDG 4 > 15.4%, these grades being equivalent to radiologist visually allocated BI-RADS density grades (Figure 1). Non-dense volume was calculated as the difference between the total and dense volume.

Two experienced specialist breast radiologists (NP and KP) also performed subjective visual estimation of the content of fibroglandular dense tissue within the breast, which was recorded according to the fourth edition BI-RADS Atlas as A = almost entirely fatty, B = scattered fibroglandular densities, C = heterogeneously dense, or D = extremely dense [17] (Figure 1).

### 2.4. Statistical Analysis

Percent and absolute breast density measurements were both log-transformed. Analyses of percent density were also adjusted for total breast volume, and the analyses of absolute density adjusted for non-dense volume. Separate multivariable regression analyses were carried out: (1) Including urban residence, (2) including working in the urban setting for those women currently working, and (3) including both urban residence and working in the urban setting. Because of the amount and pattern of missing variables, and also because of co-linearity between variables, we conducted forward stepwise regression in multivariable regression analyses, but with a generous inclusion criterion of *p* = 0.2, and with age, and BMI, and either residence or working in the urban setting always included. Multivariable regression analyses were also carried out separately for postmenopausal and pre/peri-menopausal women. *p*-values < 0.05 were considered statistically significant.

## 3. Results

### 3.1. Study Population Characteristics

Of the 1144 women, 1105 had at least one mammography attendance for which automated volumetric breast density data were available; from these, 99 women reporting previous breast cancer and a further 39 women for whom BMI could not be calculated were excluded, leaving a total of 967 included in the analysis.

Table 1 also summarizes the distribution of the categorical variables and for groups of continuous variables together with the mean and standard deviation of percent and absolute breast density. The women were aged between 36 and 81 years (median = 52 years, interquartile range (IQR) = 17 (46–63)). The great majority (94%) of women reported their ethnicity as white. The postcode of the place of work was completed by 463/967 (48%) women, of whom 378 women had data on the current place of work. Of the 967 women included in the analysis, 305 (32%) were premenopausal, 536 (55%) postmenopausal, and 100 (10%) perimenopausal.

### 3.2. Age and BMI and Breast Density

**Figure 2:**
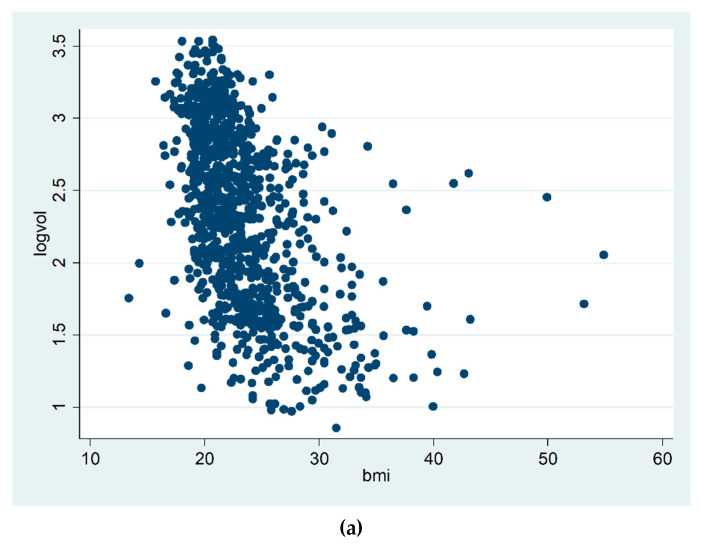

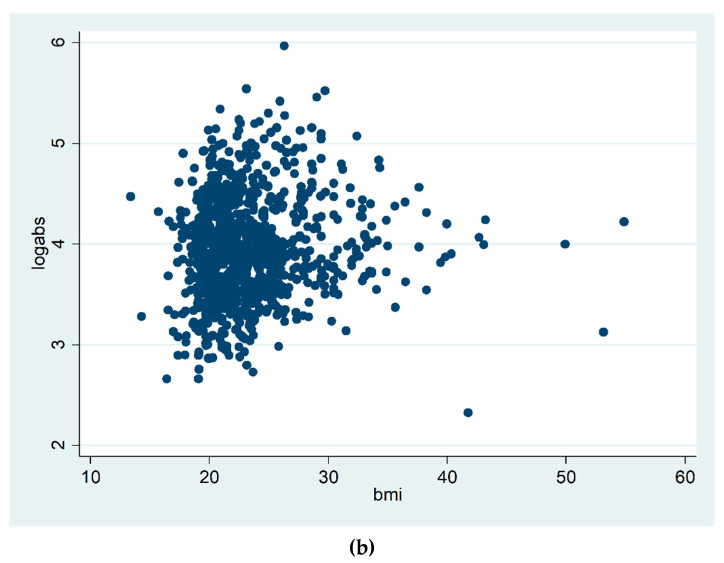
Log percent breast density by BMI (**a**). Log absolute breast density by BMI (**b**).

Increasing age was negatively correlated with both percent and absolute breast density, both alone and adjusted for BMI. Increasing BMI was negatively correlated with percent breast density but positively correlated with absolute breast density (Table 2). For women aged under 55 years, the average dense volume was 14.3 cm^3^ (SD 7.1), and average percent volumetric density was 4.2% (SD 3.9). For women aged 55 years of more, the corresponding figures were 9.6 cm^3^ (SD 6.0) and 2.5% (SD 3.1). Figure 2 shows scatter plots of BMI plotted against log percent breast density (Figure 2a), and against log absolute breast density (Figure 2b).

### 3.3. Urban Residence/Working in the Urban Setting and BMI

A total of 62.2% (602/967) of women were urban residents; only a further 25 women had previously been urban residents in London. Of the 378 women with data on the current place of work, 282 (74.6%) worked in the urban setting; only a further 6 had worked in the urban setting in the past 5 years. Of the 282 women working in the urban setting, 103 (36.5%) reported walking to work, 106 (37.6%) taking the London Underground, 60 (21.3%) taking the overground train, 104 (36.9%) travelling by car, and 56 (19.9%) travelling by bus; some women reported more than one method of transport.

Mean BMI was significantly lower in women who were urban residents compared with those who were not (23.1 vs. 23.8, *p* = 0.01) as well as in women working in the urban setting compared with those working elsewhere (23.4 vs. 25.1, *p* = 0.01).

### 3.4. Urban Residence and Breast Density

Urban residence in London was significantly associated with higher percent breast density (β = 0.10, *p* = 0.009) when analyzed alone, but this association became non-significant when BMI and age were included in the multivariable model (*p* = 0.45). In the multivariable model, increasing BMI (*p* ≤ 0.001), increasing age (*p* ≤ 0.001), and being postmenopausal (*p* ≤ 0.001) were significantly associated with lower percent breast density, whereas current HRT use (*p* = 0.01) and increased age at menarche (*p* = 0.01) were significantly associated with higher percent breast density. Detailed results for the multiple regression analysis including urban residence are shown in Table 2.

In the analysis for absolute breast density, there was no significant association between urban residence and absolute breast density. Being postmenopausal (*p* = 0.002), ever having breast fed (*p* = 0.04), and having fewer than five drinks per week (*p* = 0.002) were significantly associated with lower absolute breast density, whereas increasing BMI (*p* = 0.003) and current HRT use (*p* = 0.01) were significantly associated with higher absolute breast density.

### 3.5. Working in the Urban Setting and Breast Density

Working in the urban setting was not associated with percent breast density (B = 0.13, *p* = 0.07), either alone or when BMI and age were included (*p* = 0.45). Detailed results for the multiple regression analyses including working in the urban setting are shown in Table 3. Increasing BMI (*p* ≤ 0.001), increasing age (*p* ≤ 0.001), and being postmenopausal (*p* = 0.03) were significantly associated with lower percent breast density, while increased age at menarche (*p* = 0.04) was significantly associated with higher percent breast density.

In the analysis for absolute density, working in the urban setting was also not associated with absolute density. Increasing BMI was not significantly associated with lower absolute breast density, whereas parity (*p* = 0.01), and hysterectomy (*p* = 0.02) were significantly associated with higher absolute breast density. Analyses of postmenopausal and pre/perimenopausal women showed similar results.

### 3.6. Urban Residence and Working in the Urban Setting and Breast Density

In the multivariable regression analysis including the significant factors in Table 2 and Table 3 and including variables for both living and working in the urban setting, urban residence became significantly associated with higher percent breast density (β = 0.19, *p* = 0.002). For those living in London and still working there, the average percent breast density was 13.1 (95% CI: 12.2, 14.0), whereas for those living outside of London and/or not working there, the average was 11.3 (95% CI: 10.8, 11.8). This remained the case if the variable for working in the urban setting was excluded and the data was restricted to those women currently working. This was also the case in pre/perimenopausal women.

### 3.7. Automated Quantitative vs. Qualitative Breast Density Assessment

Percent breast density as measured by automated systems was considerably less than that estimated visually by radiologists. The correlation coefficient of the quantitatively measured VDG groupings with the subjective visual estimation according to BI-RADS was 0.80 (*p* < 0.001).

## 4. Discussion

Both BMI and MBD have been recognized as independent risk factors for breast cancer [2]. MBD is known to decrease with increasing age and BMI [5,6], and adjusting for these is important in studies of breast cancer risk and models of risk prediction based on mammographic density. [7]. In the meta-analysis by McCormack et al., studies that had adjusted for BMI had higher relative risks after this adjustment [1]. In addition to age and BMI, MBD has also been reported to be associated with lifestyle factors [8], socioeconomic status [6], and menstrual and reproductive factors [9]. Some studies have further shown an association of urban residence with increased breast density [10,11,12]. This study expands on the current knowledge of the associations of various factors, including BMI, age, lifestyle, menstrual, and reproductive factors as well as place of work and residence (urban vs. rural) with MBD.

In our study, mean BMI was significantly lower in urban women residents in London, as was hypothesized in a previous study [12]. There was no difference in the levels of physical activity reported between urban vs. rural women residents and the reasons for this merit further investigation. Our study found that being an urban resident was associated with increased percent MBD (percentage of dense tissue compared to the whole breast), but this association was not significant after adjustment for age and BMI. However, for those women currently having either an urban or rural workplace, urban residence was associated with increased percent MBD, even when adjusted for other factors. The reasons for this are unclear, but possible mechanisms might involve increased stress levels or greater exposure to environmental pollution, e.g., during travel to work, than those related to residence alone.

Previous studies have shown an association between the urban environment (either residential or workplace) with increased MBD [10,12]. However, many of these studies have not been able to account for the association of MBD with BMI. Emaus et al. showed a positive association of urbanization with percent density that persisted after adjustment for age and BMI [11]. In their study, there was no difference between median BMI in non-urbanized and extremely urbanized women. Our results showed a significant association of urban residence with percent but not absolute MBD (total volume of dense tissue expressed in milliliters). This association became non-significant when adjusted for age and BMI. While several studies have reported either a negative correlation of BMI with absolute MBD or no association [18,19] other studies have found BMI to be negatively correlated with percent MBD and positively associated with absolute MBD [20,21,22]. The study by Schetter et al. also used the same automated observer-independent volumetric MBD measurement system as in our study to measure MBD, but their study was restricted to healthy premenopausal women [20]. Restricting our study to postmenopausal women did not alter the observed associations of BMI with MBD, but it did increase the positive association of HRT use, while the effects of OCs and age at menarche became non-significant.

Current use of HRT has been shown in other studies to be associated with increased MBD, but a negative association with OC use has been less widely reported. We found no association with use of anti-estrogens, although the numbers reporting such use were small. Our finding that ever having breast fed was negatively associated with increased absolute MBD is consistent with the results of others, and indeed the decreased MBD associated with earlier menarche has also been observed elsewhere [21,22].

The results of our study highlight the importance of adjustment for age and BMI in analyses of MBD as a risk factor for breast cancer. They also contribute to the debate on whether percent or absolute density is more relevant but were not sufficient to draw firm conclusions. A study in young women at intermediate familial risk found that absolute, but not percent, MBD was a significant risk factor for breast cancer after adjusting for the area of non-dense tissue [23] and that absolute density appeared able to improve significantly the risk prediction provided by the Tyrer–Cuzick risk estimate.

Apart from factors, including BMI, age, lifestyle, menstrual, and reproductive factors, and urban vs. rural place of work and residence, there is variation as to whether the percent breast density or absolute volume of dense tissue is the most accurate predictive measure of breast cancer risk [20]. Moreover, variability in the methods of measurement, i.e., qualitative measurement by subjective radiologists’ review vs. area vs. volumetric density, may also account for some of the differences between previous studies. In this study, we used a commercially available automated observer-independent volumetric MBD measurement system and compared the quantitative MBD measurements to qualitative measurement by subjective radiologist review using BI-RADS density classifications. Percent breast density as measured by automated systems is considerably less than that estimated visually by radiologists. Nevertheless, correlation with qualitative measurement by subjective radiologist review using the BI-RADS density classification shows a high level of agreement, similar to that observed in other studies [24].While correlation does not necessarily imply agreement, this is of relevance to ensure that our findings can reasonably be compared with previous studies that used qualitative measurement by subjective radiologist review rather than automated MBD readings. The results of the current study highlight that the MBD approach is especially important when considering the incorporation of MBD measures into risk prediction models. It is also relevant to current clinical practice, with increasing use of supplementary breast ultrasound being advised in women with dense breasts reported on screening mammography and the future need to move to a more personalized screening program approach. As this has additional costs in terms of resources and reimbursement, it could be argued that such a judgement would be better made by quantitative rather than qualitative means.

The limitations of this study include the reliance on questionnaire data, with the potential for recall bias. Urban residence or working was determined based on the relevant postcodes; however, this was a fairly broad classification, and may not reflect differences between small geographical areas.

## 5. Conclusions

In conclusion, urban women in our study population had a lower BMI and our results confirm an association between residential environment and MBD. We found no association between living or working in an urban environment and increased MBD once we adequately accounted for the confounding influence of BMI and age. However, there is a possible genuine association of increased MBD in women living in an urban environment and currently working. The reasons for this are unclear but may be related to lifestyle factors or increased exposure to pollution, particularly the different air quality found between urban and rural areas, which may have a weak yet cumulative estrogenic effect [13,14,15,16,25]. Further and larger studies should be carried out to confirm these results.

## Figures and Tables

**Table 1:** Characteristics of the population sample (*n* = 967).

Variable	*n* (%)	Percent Density Mean (SD)	Absolute Density Mean (SD)
Entire population sample	967 (100)	11.9 (6.9)	60.3 (35.6)
Ethnicity			
White	912 (94.3)	11.9 (6.9)	60.0 (35.6)
Mixed	11 (1.1)	12.1 (3.3)	58.2 (36.2)
Asian	21 (2.2)	14.2 (8.3)	62.7 (39.5)
Black	3 (0.3)	12.7 (11.4)	99.4 (76.2)
Other	16 (1.6)	12.4 (6.6)	68.5 (27.0)
Missing	4 (0.4)	8.3 (5.9)	58.5 (20.8)
Education level ^1^			
None	29 (3.0)	9.4 (4.9)	56.1 (26.9)
GCSE	141 (14.6)	11.2 (7.2)	61.2 (34.8)
A level	190 (19.6)	11.2 (6.9)	61.9 (36.3)
University	351 (36.3)	12.4 (7.1)	58.7 (33.2)
Postgrad	248 (25.6)	12.9 (6.8)	61.6 (40.1)
Missing	8 (0.8)	7.3 2.2)	51.8 (11.2)
Urban Resident			
No	365 (37.7)	11.3 (6.7)	60.9 (37.8)
Yes	602 (62.2)	12.4 (7.1)	60.0 (34.3)
Urban Workplace 1A			
No	149 (15.4)	11.6 (6.7)	60.8 (36.3)
Yes	282 (29.2)	13.0 (7.1)	65.0 (39.5)
Missing	536 (55.4)	11.5 (6.1)	57.7 (33.1)
Smoker			
Current	47 (4.9)	11.8 (7.5)	56.1 (32.2)
Ex-smoker	364 (37.6)	12.2 (6.9)	61.2 (35.9)
Never	552 (37.1)	11.8 (7.0)	60.0 (35.8)
Missing	4 (0.4)	15.5 (3.9)	66.2 (30.1)
Drinks per week			
0	137 (14.2)	12.0 (7.9)	66.6 (44.6)
< 5	411 (42.5)	11.8 (6.8)	57.4 (33.5)
5–14	315 (32.6)	12.4 (7.0)	61.7 (34.4)
> 14	58 (6.0)	11.2 (6.2)	59.5 (36.0)
Missing	46 (4.8)	11.8 (5.9)	58.7 (30.4)
HRT use: current			
No	788 (81.5)	12.0 (7.0)	60.5 (36.6)
Yes	169 (17.5)	12.1 (6.9)	59.9 (31.5)
Missing	10 (1.0)	8.9 (5.3)	54.0 (22.8)
HRT use: ever			
No	645 (66.7)	12.6 (7.1)	62.0 (38.4)
Yes	319 (33)	10.7 (6.5)	56.9 (9.2)
Missing	3 (0.3)	12.6 (8.5)	61.2 (17.1)
Calcium supplement			
No	748 (77.3)	12.2 (7.1)	62.2 (37.0)
Yes	194 (20.1)	11.2 (5.9)	53.9 (29.0)
Missing	25 (2.6)	12.0 (9.2)	52.3 (35.1)
Vitamin D supplement			
No	635 (65.7)	12.0 (7.0)	62.3 (36.0)
Yes	239 (24.7)	11.7 (6.8)	55.6 (29.2)
Missing	93 (9.6)	12.3 (7.3)	58.6 (45.8)
Physical activity per week			
< 30 mins	75 (7.8)	10.8 (7.7)	65.5 (39.6)
30–60	148 (15.3)	10.4 (6.6)	61.7 (32.8)
> 60	716 (74.0)	12.4 (6.9)	59.8 (35.8)
Missing	28 (2.9)	12.2 (7.1)	52.0 (33.9)
OCs (current use)			
No	914 (94.6)	12.0 (7.0)	60.9 (3.0)
Yes	34 (3.6)	11.3 (5.3)	50.1 (28.2)
Missing	19 (2.0)	10.6 (6.0)	51.7 (28.3)
OCs (ever use)			
No	233 (24.1)	11.3 (6.9)	60.6 (36.8)
Yes	721 (74.9)	12.2 (7.0)	60.5 (35.4)
Missing	10 (1.0)	10.3 (5.2)	39.8 (10.4)
Nulliparous			
No	827 (85.5)	12.1 (6.9)	59.8 (35.4)
Yes	137 (14.2)	11.4 (7.1)	63.9 (37.0)
Missing	3 (0.3)	9.5 (4.2)	44.1 (10.2)
Menopausal status			
Pre-menopausal	305 (31.5)	14.8 (7.3)	69.2 (43.8)
Peri-menopausal	100 (10.3)	13.6 (6.5)	64.1 (37.0)
Post-menopausal	536 (55.4)	10.0 (6.1)	53.5 (27.8)
Missing	26 (2.7)	14.1 (6.7)	80.4 (36.1)
Ever breast fed			
No	308 (31.8)	10.8 (6.8)	63.3 (37.6)
Yes	600 (62.0)	12.5 (6.9)	58.6 (34.7)
Missing	59 (6.1)	13.2 (7.7)	61.8 (34.1)
Hysterectomy			
No	785 (81.2)	12.3 (7.0)	60.4 (36.4)
Yes	156 (16.1)	9.9 (6.5)	58.3 (29.5)
Missing	26 (2.7)	13.5 (6.2)	68.7 (45.4)
Family history breast cancer			
No	541 (55.9)	12.0 (7.1)	59.3 (33.2)
Yes	381 (39.4)	12.0 (6.8)	62.2 (38.7)
Missing	45 (4.6)	10.6 (6.2)	55.9 (36.0)
Age (years)			
< 45	151 (15.6)	153 (7.3)	67.4 (37.1)
45–54	381 (39.4)	13.6 (6.8)	67.2 (41.8)
55–64	254 (26.3)	9.8 (6.2)	53.5 (29.7)
65+	181 (18.7)	8.7 (5.6)	49.4 (20.5)
BMI			
< 20.6	246 (25.4)	16.3 (6.7)	55.3 (29.5)
20.6 to < 22.4	239 (24.7)	14.2 (7.3)	59.3 (29.0)
22.4 to < 25.0	249 (25.7)	10.1 (5.2)	59.3 (36.7)
≥ 25.0	233 (24.1)	7.2 (4.3)	67.7 (44.6)
Smoking (pack-years in ever smokers)			
<1	39 (11.4)	12.8 (7.3)	55.2 (32.0)
1-4	113 (32.9)	12.6 (6.4)	59.0 (32.7)
5–11	98 (28.6)	11.0 (6.5)	60.6 (34.7)
12+	93 (27.1)	11.4 (6.9)	61.9 (33.9)
Age at menarche (years)			
< 12	130 (13.4)	9.8 (6.1)	55.8 (29.8)
12	213 (22.0)	11.2 (6.9)	60.0 (34.0)
13–14	466 (48.2)	12.5 (7.1)	60.9 (39.0)
15+	143 (14.8)	13.2 (6.9)	62.3 (30.8)
Age at first birth (years)			
< 25	134 (13.9)	9.7 (6.4)	57.4 (36.5)
25–29	255 (26.4)	12.4 (7.1)	59.9 (38.7)
30–34	228 (23.6)	12.6 (6.6)	59.1 (34.1)
35+	122 (12.6)	12.9 (7.0)	63.1 (31.2)
Missing	228 (23.6)	11.7 (7.2)	62.2 (35.5)
Parity			
0	154 (15.9)	11.3 (6.9)	62.5 (35.8)
1	107 (11.1)	12.2 (7.3)	73.8 (42.0)
2	373 (38.6)	11.9 (7.1)	57.1 (31.4)
3	206 (21.3)	12.0 (6.3)	56.4 (36.7)
4+	55 (5.7)	12.7 (7.3)	61.8 (39.5)
Missing	72 (7.4)	12.8 (7.8)	61.8 (35.4)

^1^ A level is equivalent to high school graduate, University equivalent to college graduate; HRT: hormone replace therapy; OCs: oral contraceptives; SD: standard deviation.

**Table 2:** Results of multiple regression analysis including urban residence; only coefficients for those variables included in the final model are shown.

	Percent density (*n* = 917)		Absolute Density (*n* = 859)	
	Coefficient (SE)	*p*-value	Coefficient (SE)	*p*-value
Urban resident				
No	Ref		Ref	
Yes	0.02 (0.03)	0.6	−0.04 (0.04)	0.3
BMI	−0.06 (0.004)	**< 0.001**	0.009 (0.004)	0.03
Age	−0.02 (0.003)	**< 0.001**	−0.005 (0.003)	0.1
HRT current use				
No	Ref		Ref	
Yes	0.15 (0.04)	**0.01**	0.14 (0.05)	**0.01**
Age at menarche	0.04 (0.01)	**0.01**	0.02 (0.01)	0.1
Menopausal status				
Premenopausal	Ref		Ref	
Perimenopausal	−0.01 (0.06)	0.9	−0.02 (0.07)	0.8
Postmenopausal	−0.01 (0.05)	**< 0.001**	−0.19 (0.06)	**0.002**
Breast fed				
No			Ref	
Yes			−0.10 (0.05)	**0.04**
OC (ever use)				
No	Ref			
Yes	−0.06 (0.04)	0.15		
Parity			−0.03 (0.02)	0.1
Family history of breast cancer				
No			Ref	
Yes			0.05 (0.04)	0.2
Smoker				
Never			Ref	
Past			0.05 (0.04)	0.2
Current			−0.11 (0.09)	0.2
Calcium supplement				
No			Ref	
Yes			−0.08 (0.05)	0.1
Drinks per week				
0			Ref	
< 5			−0.18 (0.06)	**0.002**
5–14			−0.10 (0.06)	0.1
> 14			−0.13 (0.09)	0.2

SE: standard error.

**Table 3:** Results of multiple regression analysis including those working in the urban setting (restricted to women currently working); only coefficients for those variables included in the final model are shown.

	Percent Density (*n* = 330)		Absolute Density (*n* = 323)	
	Coefficient (SE)	*p*-value	Coefficient (SE)	*p*-value
Work London				
No	Ref		Ref	
Yes	0.06 (0.06)	0.3	0.001 (0.07)	0.99
BMI	−0.05 (0.005)	**<0.001**	0.003 (0.006)	0.6
Age	−0.02 (0.005)	**<0.001**	−0.02 (0.005)	**0.001**
HRT current use				
No	Ref		Ref	
Yes	0.11 (0.08)	0.1	0.13 (0.08)	0.1
Age at menarche	0.04 (0.02)	0.04	0.03	0.07
Menopausal status				
Pre-menopausal	Ref		Ref	
Peri-menopausal	−0.16 (0.09)	0.07	−0.12 (0.09)	0.2
Post-menopausal	−0.18 (0.08)	0.03	−0.12 (0.09)	0.2
Parity	0.04 (0.02)	0.07	−0.06 (0.02)	**0.01**
OC (ever use)				
No			Ref	
Yes			−0.11 (0.07)	0.1
Hysterectomy				
No			Ref	
Yes			0.23 (0.10)	**0.02**
Smoker				
Never			Ref	
Past			0.06 (0.06)	0.31
Current			−0.23 (0.13)	0.07

SE: standard error.
